# Orthographic and Phonological Code Activation in Deaf and Hearing Readers

**DOI:** 10.5334/joc.326

**Published:** 2024-01-30

**Authors:** Phillip J. Holcomb, Emily M. Akers, Katherine J. Midgley, Karen Emmorey

**Affiliations:** 1 Department of Psychology, San Diego State University, CA, USA; 2 School of Speech, Language and Hearing Sciences, San Diego State University, CA, USA

**Keywords:** deaf readers, ERPs, N250, N400, masked priming

## Abstract

Grainger et al. (2006) were the first to use ERP masked priming to explore the differing contributions of phonological and orthographic representations to visual word processing. Here we adapted their paradigm to examine word processing in deaf readers. We investigated whether reading-matched deaf and hearing readers (n = 36) exhibit different ERP effects associated with the activation of orthographic and phonological codes during word processing. In a visual masked priming paradigm, participants performed a go/no-go categorization task (detect an occasional animal word). Critical target words were preceded by orthographically-related (transposed letter – TL) or phonologically-related (pseudohomophone – PH) masked non-word primes were contrasted with the same target words preceded by letter substitution (control) non-words primes. Hearing readers exhibited typical N250 and N400 priming effects (greater negativity for control compared to TL or PH primed targets), and the TL and PH priming effects did not differ. For deaf readers, the N250 PH priming effect was later (250–350 ms), and they showed a reversed N250 priming effect for TL primes in this time window. The N400 TL and PH priming effects did not differ between groups. For hearing readers, those with better phonological and spelling skills showed larger early N250 PH and TL priming effects (150–250 ms). For deaf readers, those with better phonological skills showed a larger reversed TL priming effect in the late N250 window. We speculate that phonological knowledge modulates how strongly deaf readers rely on whole-word orthographic representations and/or the mapping from sublexical to lexical representations.

## 1. Introduction

The majority of deaf children in the United States begin reading instruction without the same spoken language foundation as their hearing peers, and English may be acquired more through print than through the auditory perception of speech (e.g., Goldin-Meadow & Mayberry 2001). Although phonological awareness and phonological decoding skills correlate strongly and positively with reading success for hearing readers (e.g., Hogan et al., 2005; Wagner & Torgesen, 1987), this does not appear to be the case for deaf readers. Phonological skills are not very predictive of reading ability in this population (Izzo, 2002; Mayberry et al., 2011; Sehyr & Emmorey, 2022). Nonetheless, deaf adults exhibit knowledge of spoken language phonology, which can be acquired via non-auditory mechanisms such as speech-reading and knowledge of vocal articulation (e.g., Kyle & Harris, 2006; see Musselman, 2000). For example, they perform above chance on various phonological tasks, such as rhyme judgments (Hanson & Fowler, 1987), syllable counting (Emmorey et al., 2013), and sound manipulation (Hirshorn et al., 2015), and deaf adults use a speech-based code for short-term memory of written words (Sehyr et al., 2017). Despite this phonological knowledge, however, several studies indicate that phonological codes are not automatically activated when deaf adults read words or text, in contrast to their hearing peers (Bélanger et al., 2012a, 2013; Fariña et al., 2017).

Orthographic knowledge (as assessed by spelling tasks) is positively correlated with reading skill for deaf readers – better spellers tend to be better readers (Sehyr & Emmorey, 2022). Further, better fingerspellers tend to be better readers as well (Sehyr & Emmorey, 2022; Stone et al., 2015).^1^ Some studies report faster lexical and semantic word decisions for deaf compared to hearing readers (Clark et al., 2016; Costello et al., 2021; Morford et al., 2017, 2019; Villwock et al., 2021). In addition, eye-tracking studies have found that deaf individuals read faster, skip more words, and have fewer regressions (text re-reading) compared to their reading-matched hearing peers (Bélanger et al., 2012b, 2018; Traxler et al., 2021). Based on these results, it has been argued that skilled deaf readers rely more on direct orthographic-to-semantic processing and either bypass phonological codes or do not access phonological codes as automatically or as efficiently as hearing readers (e.g., Bélanger & Rayner, 2015; Emmorey & Lee, 2021; Sehyr & Emmorey, 2022).

For hearing readers, evidence for the activation of orthographic and phonological codes during visual word recognition and the underlying neural components has come from the visual masked priming (VMP) paradigm in conjunction with event-related potentials (ERPs) first used by Grainger et al. in 2006. In the VMP paradigm, information extracted from the brief masked prime is rapidly integrated with the information extracted from the subsequent target word such that the prime and target are processed as a single perceptual event due to the blocking of recurrent neural processing by masking (Lamme et al., 1998; Lamme & Roelfsema, 2000). Using the VMP paradigm, the N250 component has been shown to be sensitive to the degree of prime-target orthographic overlap and is thought to reflect the mapping of sublexical orthography onto whole-word representations (Holcomb & Grainger, 2007; Grainger et al., 2006). In addition, the N250 component is also sensitive to phonological overlap between prime and target and is hypothesized to reflect the mapping of sublexical orthographic units to sublexical phonological codes (Grainger & Holcomb, 2009). Effects of orthographic overlap can occur with shorter prime durations and tend to appear earlier than phonological priming effects (Ferrand & Grainger, 1993; Grainger et al., 2006), suggesting that sublexical orthographic codes are initially activated and then translated into a phonological code. These codes then converge on whole-word representations which activate lexical-semantic representations, and the N400 component is sensitive to such form-meaning mappings. In sum, the N250 reflects processing at the level of form representations (orthography and phonology) while the N400 reflects processing at the level of meaning (Grainger & Holcomb, 2009).

There is mixed evidence for phonological effects on the N250 in deaf readers using the VMP paradigm. Costello et al. (2021) used a go/no-go semantic categorization task (identify an occasional animal word) and masked pseudohomophone primes to examine whether sublexical phonological codes were automatically accessed by skilled deaf readers. Pseudohomophone primes were pseudowords that sounded the same as the real word targets (e.g., *nobio* and *novio* [boyfriend]; “b” and “v” have the same pronunciation in Spanish), while control pseudowords were orthographically, but less phonologically similar to the target real words (e.g., *notio* and *novio* [boyfriend]). Costello et al. (2021) found that for hearing readers, but not for reading-matched deaf readers, masked pseudohomophone primes elicited a larger (more negative) N250 to target words (230–270 ms time window), compared to target words preceded by control primes. Note that the polarity of the phonological priming effect differs from previous studies which find that pseudohomophone primes *reduce* the N250 amplitude, as typical for priming effects (Grainger et al., 2006; Eddy et al., 2016). The lack of N250 phonological effects for deaf readers is consistent with the behavioral results of Bélanger et al. (2012a) who found no phonological effects for French deaf readers performing a lexical decision task with masked phonologically-related compared to orthographically-related primes.

In contrast, Gutierrez-Sigut et al. (2017) found evidence of automatic phonological code activation in deaf readers of Spanish when they performed a lexical decision task with masked pseudohomophone and orthographic control primes. In this case, the more typical pattern was found in which the amplitude of the N250 was reduced for target words with pseudohomophone primes compared to control primes for both deaf and hearing readers. In addition, lexical decision times were faster for targets with pseudohomophone than control primes for both groups, consistent with some studies reporting (limited) sensitivity to pseudohomophones in deaf readers (Transler & Reitsma, 2005; Friesen & Joannise, 2012). The Gutierrez-Sigut et al. (2017) and Costello et al. (2021) studies differed in a number of ways. Gutierrez-Sigut et al. (2017) tested less-skilled deaf readers and used a more sensitive masked priming paradigm (“sandwich” priming; Lupker & Davis, 2009). Either of these factors could impact whether the N250 is sensitive to phonological manipulations in deaf readers.

Transposed letter (TL) paradigms have been used to assess orthographic precision and the sensitivity of the N250 to sublexical orthographic processing. Masked TL primes (e.g., chikcen – CHICKEN) elicit faster lexical decision times compared to letter substitution primes (e.g., chidven – CHICKEN), and this priming effect is argued to indicate that letter position coding in sublexical orthographic representations is flexible, i.e., not precise (Perea & Lupker, 2004; Meade et al., 2020). Further, Perea and Carreiras (2006) showed that masked TL priming is driven by orthographic, not phonological representations (see also Perea & Carreiras, 2008). They compared the effects of TL primes in Spanish (e.g., relovucion – REVOLUCIÓN) with effects of pseudohomophone TL primes (e.g., relobucion- REVOLUCIÓN) and orthographic control primes (e.g., reloducion- REVOLUCIÓN). The amount of masked TL priming was independent of pronunciation, with similar TL priming effects for both the pseudohomophone and the non-homophone TL primes (compared to control primes). Finally, masked TL primes elicit reduced N250 amplitudes compared to orthographic control primes (Carreiras, Vergara, et al., 2009), and this effect occurs earlier than pseudohomophone priming effects, suggesting that orthographic sublexical processing precedes access to phonological codes (Grainger et al., 2006; Carreiras, Perea, et al., 2009).

Deaf readers have been shown to be sensitive to TL effects, exhibiting longer lexical decision times for TL nonwords than control nonwords (Fariña et al., 2017) and exhibiting TL masked priming effects – faster response times for target words with TL than control primes (Meade et al., 2020). In an unprimed, masked lexical decision study, Lee et al. (2022) found that deaf readers were faster and more accurate at rejecting TL nonwords compared to hearing readers, possibly due to more direct orthographic-to-semantic processing and/or differences in early visual orthographic processing (see also Gutierrez-Sigut et al., 2022). However, there were no differences in the N250 amplitudes for the two groups (larger negativity for nonwords than words). Similarly, Meade et al. (2020) found that TL ERP priming effects (reduced N250 amplitudes for targets with masked TL primes than control primes) were largely the same for skill-matched deaf and hearing readers. Together, these results suggest that the activation of orthographic codes is similar for deaf and hearing readers, despite differences in phonological skills (see also Meade et al., 2019).

For hearing readers, both pseudohomophone and TL masked primes reduce the N400 amplitude for target words, which is argued to reflect the interaction between lexical-semantic representations and both whole-word phonological representations and whole-word orthographic representations (Holcomb & Grainger, 2007). Similar TL effects on the N400 component for deaf and hearing readers were reported by Meade et al. (2020) and Lee et al. (2022). However, reports of pseudohomophone effects on the N400 for deaf readers are mixed. Gutierrez-Sigut et al. (2017) observed an N400 priming effect with masked pseudohomophone primes for deaf and hearing readers. In addition, the size of the N400 effect (but not the N250 effect) was correlated with phonological skill (assessed by a syllable counting task) and reading ability for the deaf readers, but not for the hearing readers. This result suggests that reading skill and phonological knowledge may strengthen whole-word phonological representations for deaf readers, whereas for hearing readers these skills strengthen sub-lexical phonological representations – correlations were observed for the N250, not the N400 priming effect for the hearing group.

However, in an (unprimed) lexical decision ERP experiment, Costello et al. (2021) found that for deaf readers, the N400 amplitude was not modulated by the whether the nonword was a pseudohomophone or a control pseudoword, whereas for hearing readers, pseudohomophones elicited a reduced N400 compared to control pseudowords, indicating that whole-word phonological representations of real words were activated by their related pseudohomophones. Hearing readers also made many more errors on pseudohomophones (accepting them as real words) compared to the deaf readers who were equally accurate at rejecting both pseudohomophones and control pseudowords. One possible explanation for the discrepancy between the findings of Gutierrez-Sigut et al. (2017) and Costello et al. (2021) is that for deaf readers phonological effects may be dampened when pseudohomophones are targets, rather than primes for real words. Deaf readers may be better able than hearing readers to strategically suppress phonological processing when performing the lexical decision task. In contrast, masked pseudohomophone primes are processed subconsciously and are not subject to such strategic effects.

### 1.1. The present study

Given the conflicting results from previous studies on pseudohomophone priming in deaf readers and the lack of comparative orthographic priming effects between deaf and hearing readers the goal of this study was to examine the time course of activation for orthographic and phonological codes in skilled deaf readers using ERPs and the visual masked priming paradigm. We manipulated the relationship between masked prime letter strings and subsequent five-letter target words. The critical prime stimuli were all non-words and could either be composed of the same letters as the target but with two letters reversed (TL primes), e.g., *toast* – *tosat*, or the nonword primes could have the same pronunciation as the target (pseudohomophone or PH primes), e.g., *brain* – *brane*. TL and PH primes were contrasted with matched control primes where two critical letters of the TL or one letter of the PH primes were substituted, e.g., tosat – *toret* and *brane* – *brant*.

**Figure 1:**
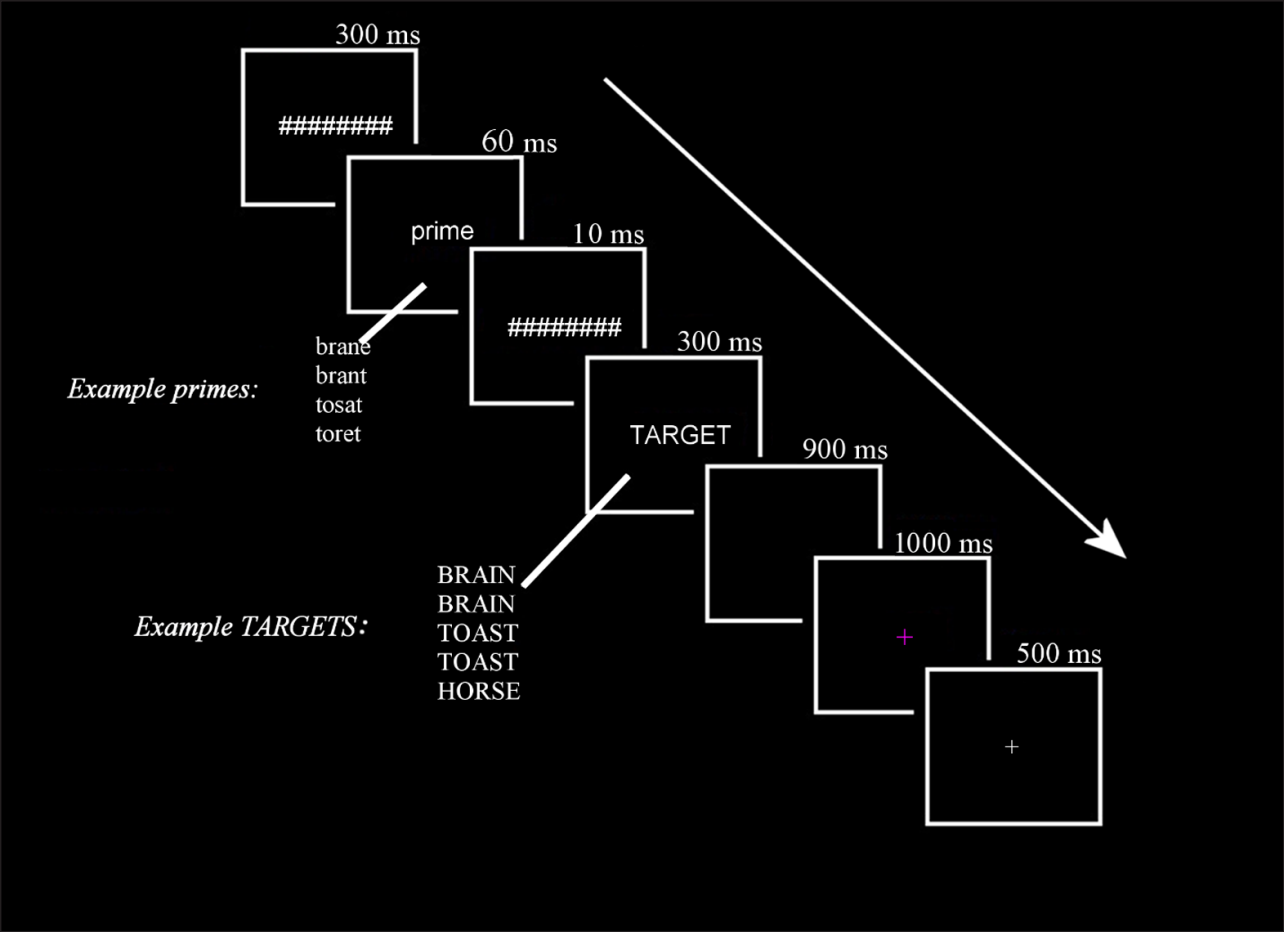
Schematic of trial timing with examples of the four prime and five target conditions.

We measured the ERPs generated by target words in deaf and hearing adult readers who were matched on overall reading ability. Following the seminal study of Grainger et al. (2006), ERPs on each trial were recorded to a series of visual stimuli displayed in rapid succession; this included a forward mask (a row of hash marks) presented for 300 ms, a prime word presented in lowercase letters for 60 ms, a backward mask (the same hash marks) for 10 ms and a target word in all uppercase letters for 300 ms (see Figure 1). Participants engaged in a go/no-go semantic categorization task in which they were told to press a single button whenever they saw occasional probe words that named an animal (~15% of trials). The remaining non-probes (so-called critical trials) contained the experimental manipulations of pseudohomophone (e.g., brane-BRAIN) vs. letter substitution control (e.g., brant-BRAIN) priming and transposed letter (e.g., tosat-TOAST) vs. letter substitution (e.g., toret-TOAST) priming.

We predicted that for hearing readers, both TL and PH primes would reduce the N250 amplitude for target words compared to control primes, but this modulation would occur earlier for TL than for PH primes as previously reported by Grainger et al. (2006). For deaf readers, we predicted similar N250 priming effects for TL primes as for hearing readers, but possibly with an earlier onset for deaf readers (see Winsler et al., 2023). Given the mixed results for pseudohomophone priming in deaf readers, it was not clear whether we would observe PH priming on either the N250 or N400 components for skilled deaf readers. If PH priming were observed for deaf readers, we expected that the effect would be delayed compared to hearing readers who are likely to activate sub-lexical phonological representations more automatically and efficiently. In addition, we investigated whether the size of any observed priming effects was correlated with reading skill, spelling skill (a measure of orthographic precision), or phonological skill.

## 2. Methods

### 2.1. Participants

A total of 54 volunteers participated in this experiment but of these, 18 were not included in analyses because of equipment failure or excessive EEG artifact. Of the remaining 36, 18 were congenitally deaf adults (7 female; mean age = 32 years, SD = 7.76, range = 18–52 years) who were either native signers of ASL (born into deaf signing families; N = 16) or acquired sign language before age five (N = 2). The other 18 participants were hearing adults (10 female; mean age=28 years, SD = 7.7, range = 18–50 years) who were native speakers of English (none knew ASL). The deaf participants were severely to profoundly deaf (db loss ≥ 70 db), and all were congenitally or prelingually deaf. The mean number of years of education for the deaf participants was 17.8 (SD = 2.21) and for the hearing participants, it was 15.06 years (SD = 2.44). All participants had normal or corrected to normal vision. One deaf and one hearing participant was left-handed. The protocol for this study was approved by the San Diego State University Internal Review Board (IRB) which also approved the consent form signed by each participant. Participants signed the consent form only after being told in writing as well as verbally or via ASL about the details of the experiment. Both a native ASL signer and hearing experimenter were on hand for the consent process and the experimental run.

### 2.2. Behavioral tests

All participants underwent an assessment battery that measured reading comprehension (Passage comprehension subtest of the Woodcock Johnson IV; Schrank et al., 2014), spelling recognition (Andrews & Hersch, 2010), and phonological awareness (Hirshorn et al., 2015). The phonological awareness test consisted of two subtests: an “odd man out” sound test (which of three pictures has a different first sound or a different vowel?) and a sound manipulation test: combine the first sound of one picture (e.g., a bird) and the rime of the second picture (e.g., a toe) to create a new word (e.g., bow) which is typed on a keyboard. As shown in Table 1, the deaf and hearing readers did not differ significantly in reading comprehension or spelling ability (although deaf readers were marginally more accurate on spelling recognition). The hearing readers were significantly more accurate than the deaf readers on the two phonological awareness tests; note that the deaf readers scored quite a bit above chance (.33).

**Table 1:** Assessment scores for deaf and hearing participants *M (SD)*.


	WJ PASSAGECOMPREHENSION	SPELLING RECOGNITION	PHONOLOGICAL ABILITY SOUNDS TEST	PHONOLOGICAL ABILITY MANIPULATION TEST

Deaf	35.9 (5.23)	74.2 (8.42)	.65 (.12)	.63 (.19)

Hearing	38.7 (3.06)	71.8 (9.86)	.88 (.14)	.87 (.10)

	*p* = .44	*p* = .06	*p* < .0001	*p* < .0001


### 2.3. ERP stimuli

The critical stimuli for this experiment were formed from a master list of 200 five-letter words where we changed or transposed two internal letters to form five-letter pseudohomophones (PH), and transposed letter (TL) nonwords. The final 160 nonword stimuli were selected based on a pre-experiment behavioral study, in which 12 hearing participants were asked to verbally name the real word from which the PH or TL nonwords were formed. Mean naming RTs for PH nonwords was 623 ms (SD = 74 ms) and for TLs, which involved unscrambling and then naming, was 900 ms (SD = 128 ms). We narrowed the list to the final critical items by first removing PH items exceeding 800 ms naming latency and TL items exceeding 1000 ms. We then chose the 80 most accurately identified items in each condition. In the PH condition mean accuracy for the final 80 items was 97% (SD = 9) and in the TL condition it was 95% (SD = 10). For each of the 80 items in the PH condition we then formed an alternative pronounceable control nonword that was not a pseudohomophone by substituting one letter in the root pseudohomophone (e.g., brain > brane > brant). Likewise, we formed control TL pronounceable nonwords by substituting the two transposed letters with other letters (e.g., toast > tosat > toret).

The 160 critical items were arranged in pairs, and the first member of each pair, which was always a nonword, was referred to as the prime and the second member, which was always a word, was referred to as the target. From these pairs four stimulus lists were formed. In each list there were two conditions (related vs. control) and two priming types (PH vs.TL) with 40 items per condition. Note that the same target words occurred an equal number of times in both related and control conditions across lists assuring that priming effects within conditions were always based on the same target word.

Each list also contained 30 trials where an animal probe name appeared in the target position and 10 trials where an animal probe appeared in the prime position. Animal probes were used as “go” items in a go/no-go semantic categorization task in which participants were instructed to rapidly press a single button with their right thumb whenever they detected an animal probe name. Participants were told to read all other words passively without responding (i.e., critical stimuli did not require an overt response). The 10 probe items appearing in the prime position served as a measure of prime detectability, thus providing an objective measure of the effectiveness of the masking procedure. Prior to the experimental run, a practice block was run to familiarize the participant with the procedure.

All stimuli were presented in the center of a 24-inch gaming LCD monitor set to a refresh rate of 100Hz and located approximately 125 cm directly in front of the participant. Stimuli were displayed at high contrast as white letters (Courier fixed font) on a black background. Each trial (see Figure 1) consisted of a forward mask of seven-pound signs (#######) presented for a duration of 300 ms and was immediately followed by a five letter, lower case 60 ms prime letter string. The prime was followed by a backward mask of seven-pound signs (#######) with a duration of 10 ms which was in turn replaced by a five letter, uppercase 300 ms target word. Targets were followed by a 900 ms black screen (allowing ERPs to be collected) and a trial ending purple fixation cross (1000 ms). The purple cross cued the participant to blink if necessary. A white fixation cross (500 ms) alerted the participant that the next trial was about to begin. All word stimuli were presented within the fovea (less than 2° of horizontal and 1° of vertical visual angle).

### 2.4. EEG recording procedure

**Figure 2:**
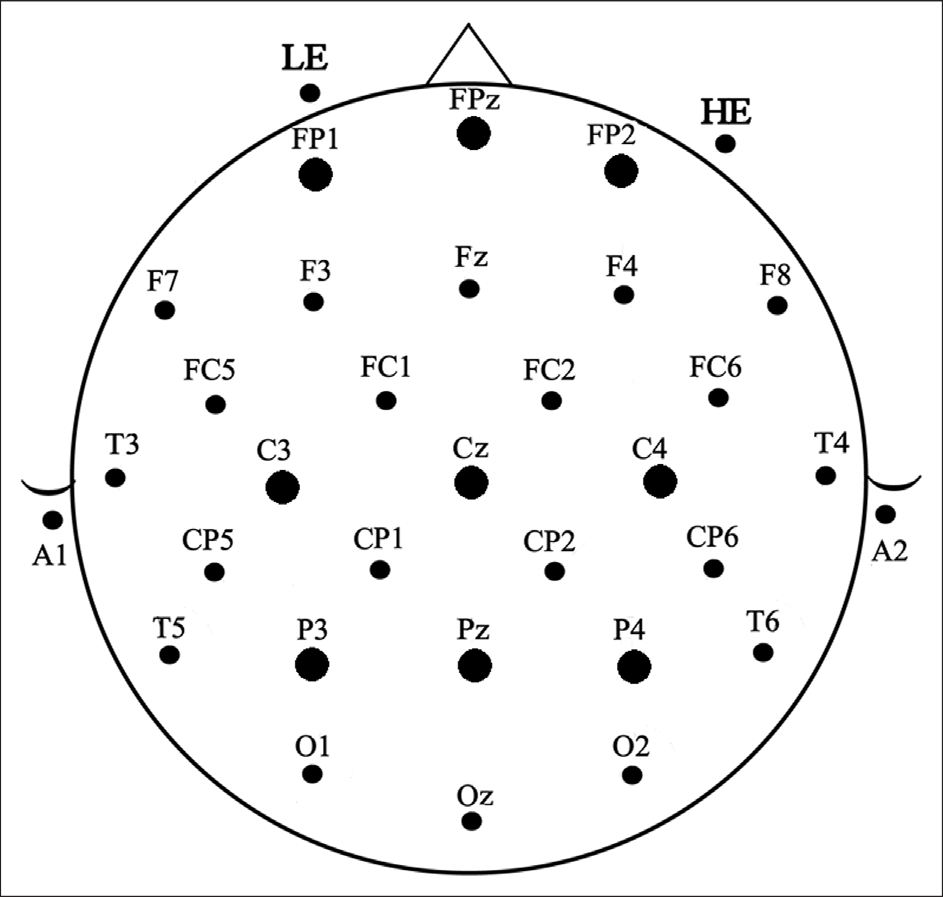
32 channel electrode montage including sites below the left eye (lower eye – LE) and to the right of the right eye (horizontal eye – HE). The nine ANOVA analysis sites are indicated with larger black dots.

Participants were seated in a comfortable chair in a sound attenuated, darkened room. An electro-cap fitted with tin electrodes was used to record continuous EEG from 29 sites on the scalp including sites over left and right fronto-polar (FP1/FP2), frontal (F3/F4, F7/F8), frontal-central (FC1/FC2, FC5/FC6), central (C3/C4), temporal (T5/T6, T3/T4), central-parietal (CP1/CP2, CP5/CP6), parietal (P3/P4), and occipital (O1/O2) areas and five midline sites over the frontal pole (FPz), frontal (Fz), central (Cz), parietal (Pz) and occipital (Oz) areas (see Figure 2). Four additional electrodes were attached: one below the left eye (to monitor for vertical eye movement/blinks – LE), one to the right of the right eye (to monitor for horizontal eye movements – HE), one over the left mastoid (reference) and one over the right mastoid (recorded actively to monitor for differential mastoid activity). All EEG electrode impedances were maintained below 2.5 kΩ (impedance for eye electrodes was less than 5 kΩ). The EEG was amplified by a NeuroScan Synamps 2 system with a bandpass of DC to 200 Hz and the EEG were continuously sampled at a rate of 500 Hz.

### 2.5. Data analysis

Prior to averaging the EEG data blink artifacts were removed using ICA as recommend by Jung et al. (2000). Single trial EEG data time-locked to a point 100 ms pre-target onset and continuing for 700 ms were averaged at each of the 29 scalp electrode sites for each of the four priming conditions. The resulting ERPs were baselined to the average of the 100 ms pre-target period and digitally bandpass filtered between .01 and 15 Hz. Only trials without residual EEG artifact were included in the averages. On average 5.5% of trials were rejected after ICA due to artifact. Due to differential mastoid activity in the grand average waves, offline the ERP data were re-referenced to the average of the two mastoids. In order to carefully quantify the time course of the ERP effects, following Grainger et al. (2006) we measured mean amplitudes in three contiguous windows after target onset: 150 through 250 ms, 250 through 350 ms, and 350 through 550 ms. Mixed design analyses of variance (ANOVAs) with one between-subject factor of reading Group (Deaf vs. Hearing) and four within-participants factors of prime Type (pseudohomophone vs. TL), Priming (related vs. control) and two scalp distribution factors (Laterality and Ant-Post) were used to analyze the ERP data. The latter two variables were calculated from three anterior (FP1, FPz, FP2), three middle (C3, Cz, C4), and three posterior (P3, Pz, P4) electrode sites as in Grainger et al. (2006). The Geisser and Greenhouse (1959) correction was applied to all repeated measures with more than one degree of freedom (corrected p values are reported). Significant interactions between Group and Priming were followed-up with separate within-subject ANOVAs on the two groups separately. Note that while we used factorial ANOVAs, to mitigate against the problems of explosive familywise inflation of alpha (Luck & Gaspelin, 2017), we only consider effects that involve the Priming variable (control vs. related) and its interactions with Type of prime and scalp distribution factors.

## 3. Results

### 3.1. ERPs

**Figure 3:**
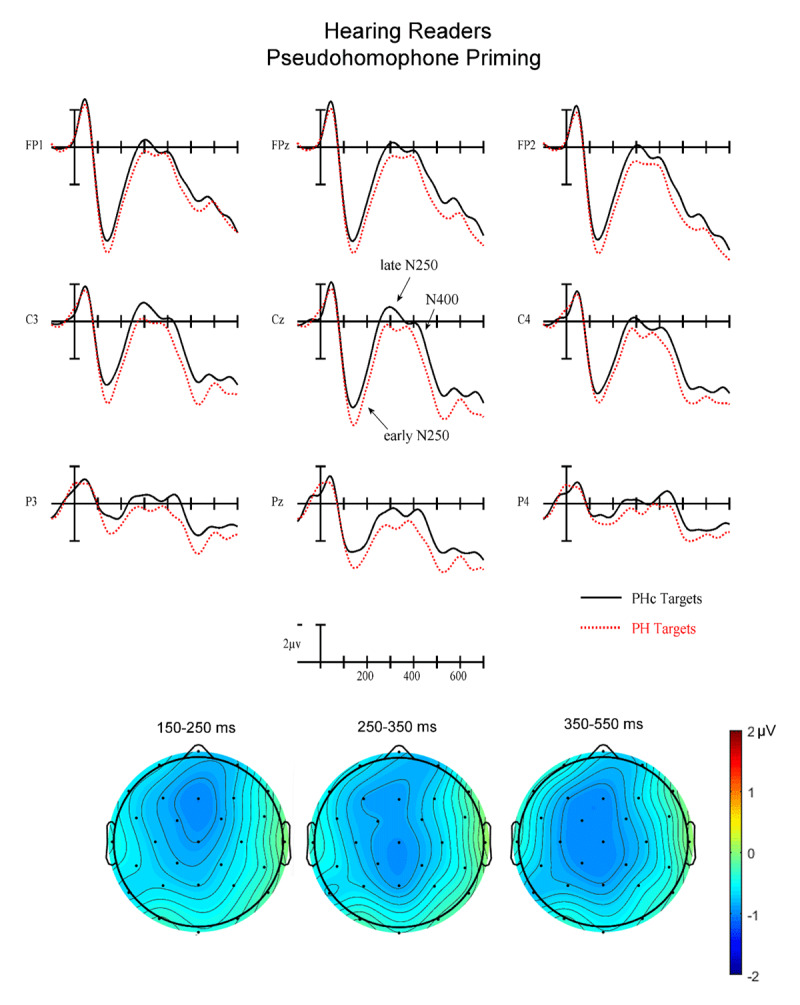
(Top) Grand average ERPs from 9 analysis electrodes time-locked to target words for the Pseudohomophone (PH) (red dotted) and letter substitution control (PHc) (solid black) conditions in hearing readers. In this and subsequent figures target onset is the vertical calibration bar and negative is plotted up. (Bottom) voltage maps showing the priming effect (Control-PH) in each of the three analysis epochs.

**Figure 4:**
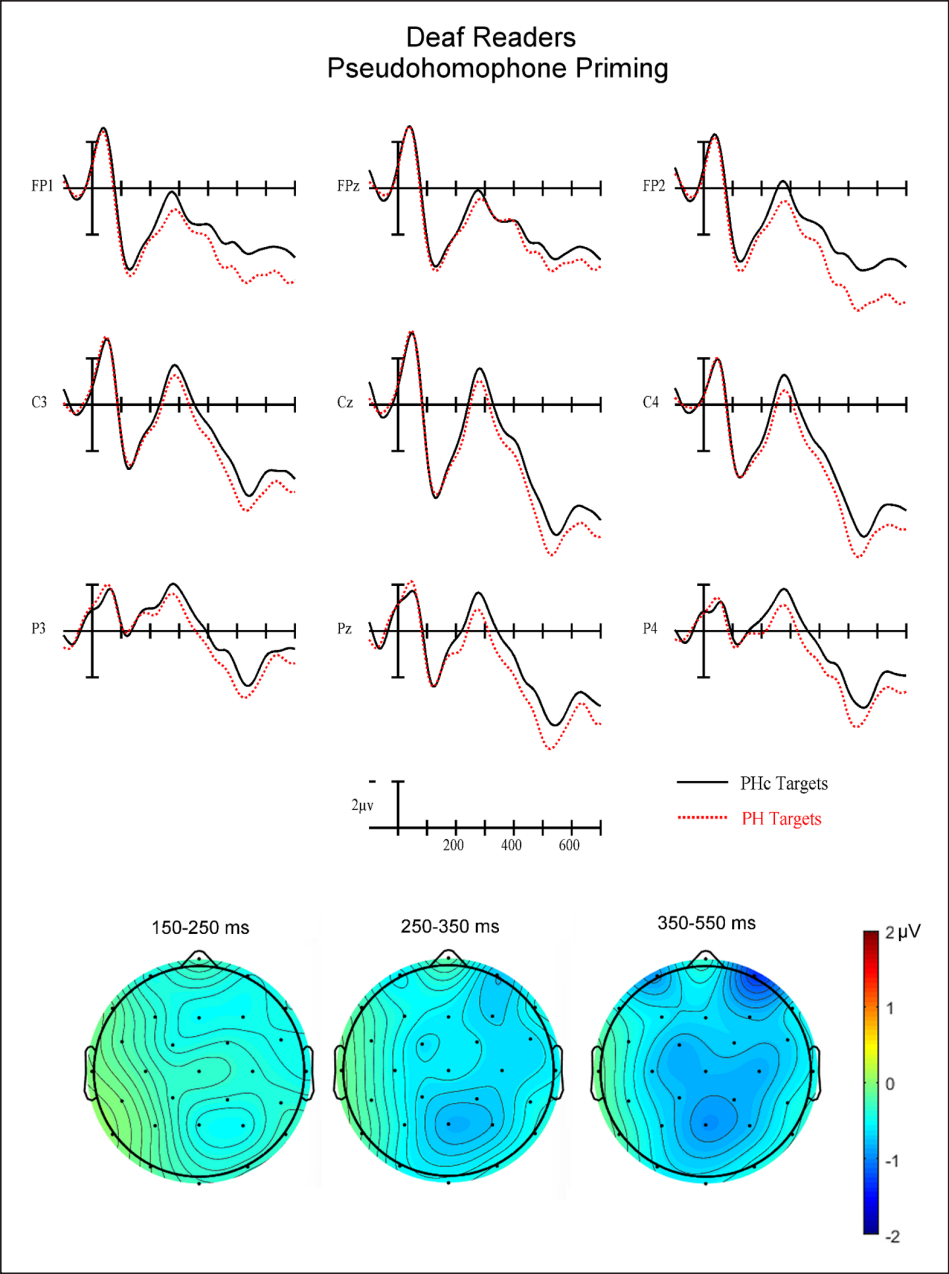
(Top) Grand average ERPs from 9 analysis electrodes time-locked to target words for the Pseudohomophone (PH) (red dotted) and letter substitution control (PHc) (solid black) conditions in deaf readers. (Bottom) voltage maps showing the priming effect (Control-PH) in each of the three analysis epochs.

**Figure 5:**
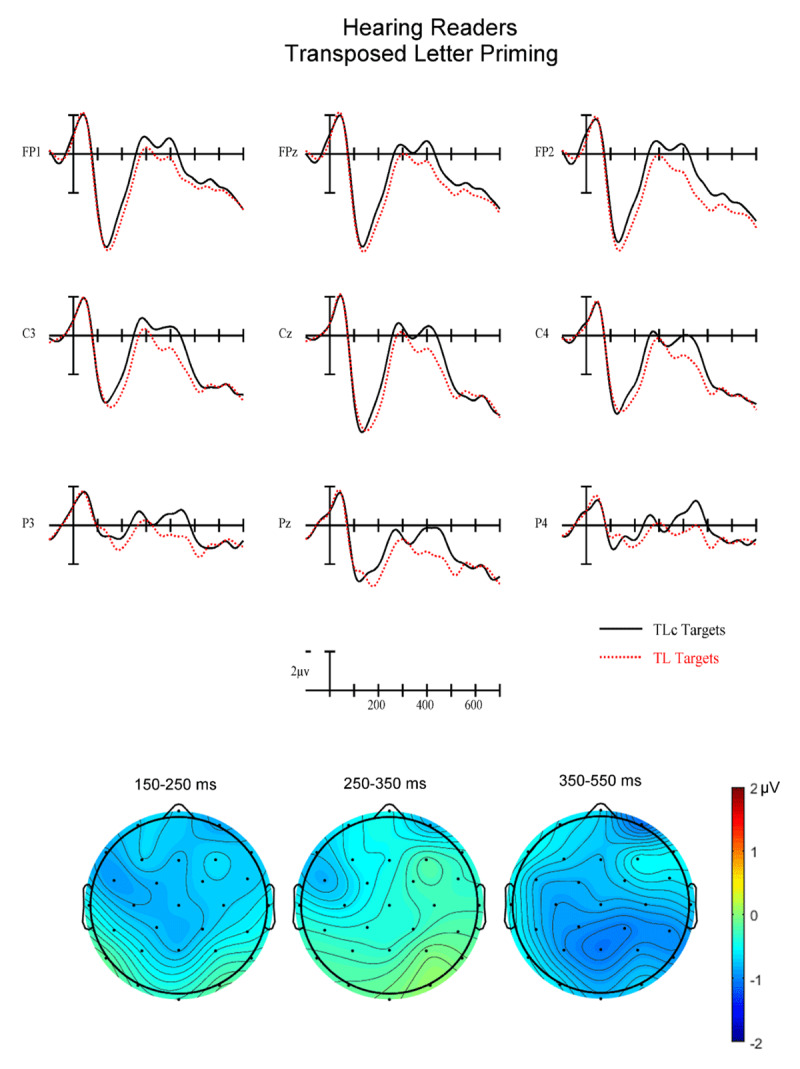
(Top) Grand average ERPs from 9 analysis electrodes time-locked to target words for the Transposed Letter (TL) (red dotted) and letter substitution control (TLc) (solid black) conditions in hearing readers.

**Figure 6:**
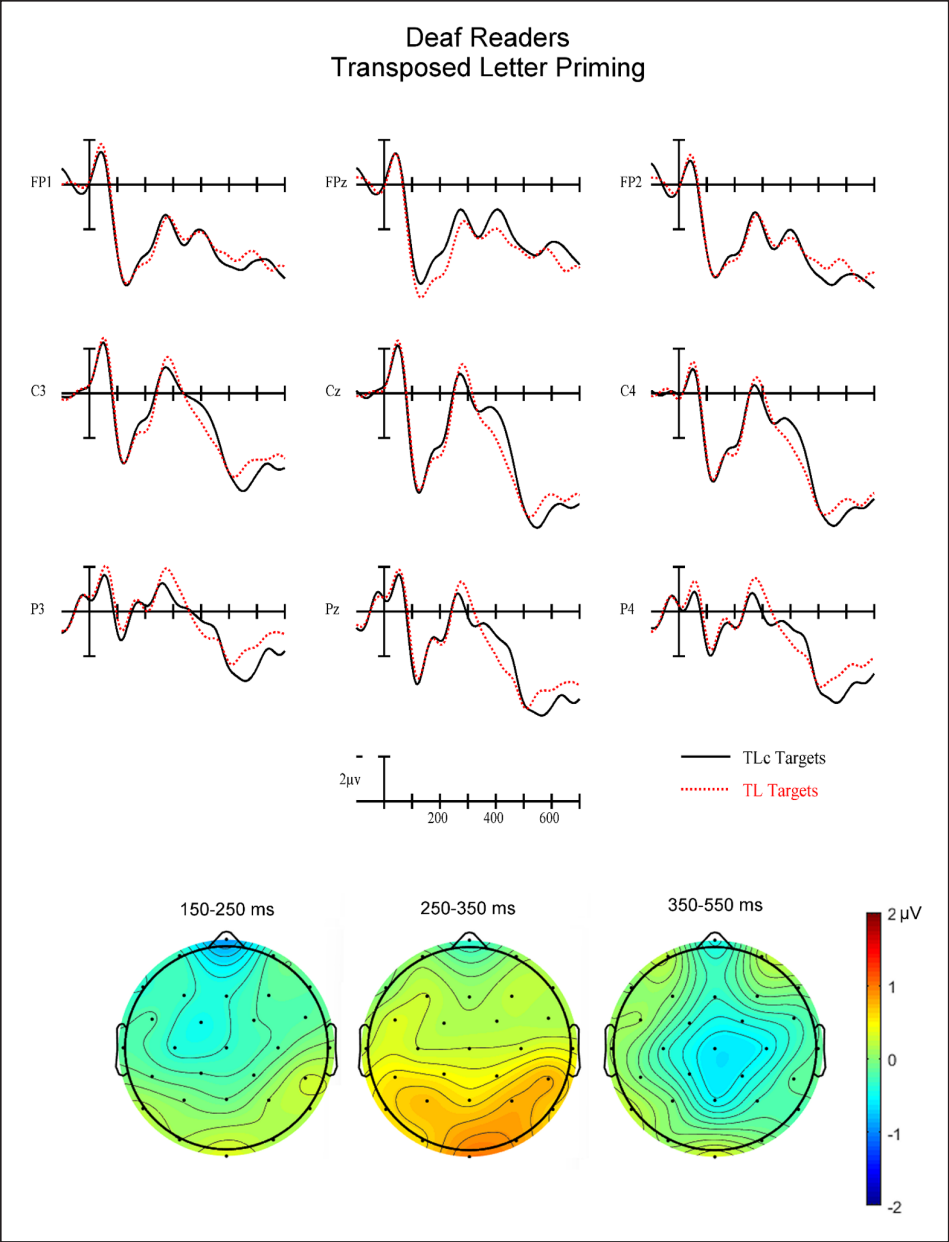
(Top) Grand average ERPs from 9 analysis electrodes time-locked to target words for the Transposed Letter (TL) (red dotted) and letter substitution control (TLc) (solid black) conditions in deaf readers.

Plotted in Figures 3, 4, 5, 6 are the ERPs contrasting the related and control targets for pseudohomophone and TL priming for the two reading groups. Keep in mind when viewing these wave forms that while we time-locked to target word onset these ERPs are actually an amalgamation of brain activity resulting from presentation of the target itself as well as the immediately preceding stimuli -- most notably the prime at –70 ms and backward mask at –10 ms (e.g., note the negativity at 50 ms which is too early to be the N1 to the target and likely is a summation of prime and backward mask N1s). For this reason, it is important to focus on the differences between related and control conditions (i.e., priming effects) where the pre-target stimuli have been carefully equated and therefore are likely to be well controlled. As can be seen, there are small priming effects starting around 150 ms (early N250 epoch) and continuing through to 550 ms (N400 epoch).

**Early N250 Epoch (150–250 ms)**. In this first epoch there was a significant main effect of Priming (F(1,34) = 9.35, p = .0043) with control target words generating greater early N250 activity (1.34 µv) than related target words (1.80 µv). There was also a marginal Group × Priming interaction (F(1,34) = 3.66, p = .064). To better understand this trend in the data we conducted follow-up ANOVAs in each reading group separately. While the hearing readers produced a robust overall priming effect in this epoch (F(1,17) = 10.69, p = .0045) with greater negativities for control targets following unrelated primes compared to related primes, deaf readers did not show evidence of a significant Priming effect (all ps < .37 – see the left most voltage maps in Figures 4 and 6). Neither group showed differences for the two Types of priming (PH vs. TL) in this epoch (all interactions between Priming and Type of prime ps > .25).

**Late N250 Epoch (250–350 ms)**. In this epoch there was again a main effect of Priming (F(1.34) = 10.48, p = .003) with control stimuli producing more negative-going ERPs than related (–.21 vs. .17 µv). There was also a Group × Priming interaction (F(1,34) = 7.68, p = .009). We again conducted follow-up ANOVAs in each reading group separately. The hearing group produced a significant main effect of Priming (F(1,17) = 19.39) p = .0004) but did not reveal a significant interaction between Priming and Type of prime (ps > .3). In both cases the unrelated condition produced more negative-going ERPs in this epoch. The deaf group did not produce a significant main effect of Priming (ps > .33) but did generate a significant three-way interaction between Priming, Type of prime and AntPost distribution (F(2,34) = 4.42, p = .02). As can be seen by comparing the center voltage maps in Figures 4 and 6, the PH priming effect has a clear N250-like broadly distributed negativity. However, the TL priming effect has a *reversed* N250 effect with more negative-going ERPs at posterior sites for targets following TL primes (–1.34 µv) compared to targets following control primes (–0.63 µv).

**N400 Epoch (350–550 ms)**. In this final epoch there was again a main effect of Priming (F(1,34) = 13.82, p = .0007) with greater negativity for targets following control (1.62 µv) than related (2.24 µv) primes (see Figures 2, 3, 4, 5, 6). However, there were no interactions between Priming and Type of priming (all ps > .2), Priming and reading Group (all ps > .18) or any of the three-way interactions between Group, Priming and Type (all ps > .18).

### 3.2. Summary

Across two of the three analysis epochs (early N250 and late N250), hearing and deaf readers produced different patterns of orthographic (TL) and phonological (PH) masked priming. While hearing readers revealed robust early and late N250 priming effects for both TL and PH conditions (control primes more negative-going than related) as well as later N400 priming, deaf readers showed this pattern only in the N400 window. For the early N250 (150–250 ms) deaf readers did not produce an obvious priming effect for either TL or PH primes. However, in the late N250 window (250–350 ms) deaf readers produced a similar priming effect as seen in the hearing readers for PH priming but a reversed priming effect for TL primes (greater negativity to related TL primes then for TL control primes). In the N400 epoch, both PH and TL primes produced similar priming effects for both deaf and hearing readers.

### 3.3. Correlations

Although all 36 participants were competent readers, both groups exhibited a range of reading related skills (see Table 1) which allowed us to perform correlations between the mean amplitude of the ERP priming effects (PH and TL) in all three temporal epochs (early N250, late N250 and N400) and measures of language skill from three behavioral tests. These included overall reading skill (Woodcock-Johnson IV – passage comprehension; Schrank et al., 2014), spelling skill (Andrews & Hersch, 2010), and phonological skill (combined subtest scores from Hirshorn et al., 2015). Only correlations surviving FDR correction are reported (Groppe et al., 2011).

**Figure 7:**
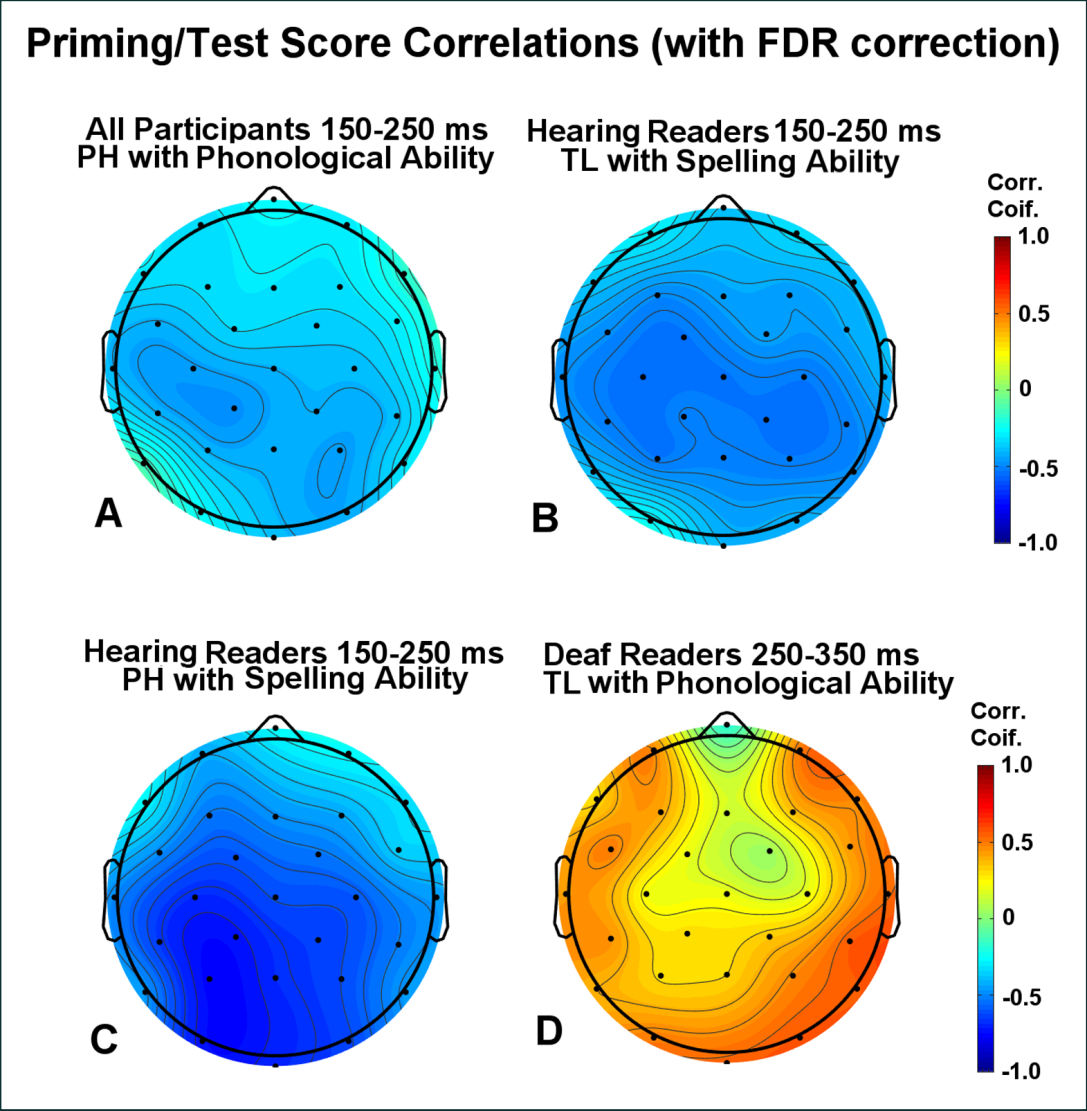
Correlation maps formed by plotting r values calculated between the priming effect at each scalp electrode with language test scores for each participant. **(A)** the correlation map across all 36 participants between phonological scores and mean amplitude PH priming effects from 150–250 ms (early N250). **(B)** correlation map for hearing readers between spelling scores and TL priming effects from 150–250 ms (early N250). **(C)** correlation map for hearing readers between spelling scores and PH priming effects from 150–250 ms (early N250). **(D)** correlation map for deaf readers between phonological scores and TL priming effects from 250–350 ms (late N250).

The only significant correlation across the two groups was the relationship between phonological skill and pseudohomophone priming in the early N250 epoch (150–250ms – see Figure 7A). Better phonological scores were associated with stronger early pseudohomophone priming. In correlations run separately for the two groups, there were significant correlations between spelling scores and both early PH and TL priming in the hearing group (see Figure 7B and C). Better spellers tended to have larger early priming for both TL and PH items. None of the correlations between test scores and ERP priming survived FDR correction in the deaf group.

In a final set of correlation analyses, we sought to better understand the significant reversed TL priming effect for deaf readers in the late N250 epoch (250–350 ms). Here we correlated scores on each of the three language tests with the mean late N250 amplitude across the four electrode sites with the largest reversed priming effect (Oz, O2, P4 and CP4 – see Figure 6 bottom center). While all three tests produced positive correlations with mean N250 amplitude, only the phonological test scores resulted in a significant effect (r = .57, p = .039 after correction) indicating that deaf readers with greater phonological ability produced larger reversed late N250 effects for TL primes (see Figure 7D). There were no significant correlations with test scores in this epoch for the hearing readers (all ps > .2) although, as reported above, there were a number of significant negative correlations between spelling ability and the early N250 window (150–250 ms). Here the correlations indicated that better spelling ability in hearing readers was associated with a larger (i.e., more negative) early N250 effect for TL primes (see Figure 7B).

## 4. Discussion

As we predicted for hearing readers, both transposed letter (TL) and pseudohomophone (PH) primes reduced the amplitude of the N250 compared to their respective control primes, reflecting this component’s sensitivity to sublexical orthographic and phonological structure. However, rather than occurring earlier, TL priming effects occurred simultaneously with PH priming effects – a result that contrasts with the original Grainger et al. (2006) findings. However, Eddy et al. (2016) also reported similar onsets (starting around 150 ms) for TL and PH priming in hearing children, ages 8 – 10 years. Unlike the participants in the Grainger et al. (2006) study who were all young undergraduates at Tufts University, our participants were generally older (mean age = 28 years) and most were from the community and were not students. It is possible that TL priming effects only precede PH priming effects for highly-skilled, young adult readers who may activate orthographic codes very quickly. This possibility could be fertile ground for future individual difference studies using the Grainger et al. (2006) paradigm.

During the early N250 epoch (150–250 ms), hearing readers showed TL and PH priming effects, but the deaf readers did not show clear evidence of priming effects until the late N250 epoch (250–350 ms). This result supports our prediction that PH priming would be delayed for deaf readers because activation of phonological codes is likely to be less robust and/or less automatic compared to hearing readers. Across the two groups, those with better phonological skills exhibited a larger PH priming effect in the early N250 epoch, but this correlation appears to have been largely driven by the hearing readers as there was no clear effect of PH priming in the early N250 for deaf readers. In addition, for the hearing group only, better spellers exhibited larger early N250 priming for both PH and TL items. These findings for the hearing readers suggest a link between the preciseness of sublexical phonological/orthographic representations and the amplitude of sublexical priming indexed by the N250 component.

However, the TL N250 priming results for deaf readers was surprising for two reasons: 1) the TL effect occurred later (not earlier) compared to hearing readers and 2) TL priming was reversed, such that target words with TL primes exhibited significantly *greater* negativity than targets with TL control primes. The reversed N250 TL priming also contrasts with the typical TL priming reported by Meade et al. (2020) for a group of similar deaf readers. In contrast to our study, Meade et al. (2020) used a sandwich priming paradigm in which the target word was briefly presented (30 ms) before the TL or control prime. This paradigm is argued to boost sublexical orthographic priming effects by reducing lexical competition from orthographic neighbors that can suppress the activation of the target word (Lupker & Davis, 2009). That is, presentation of the target word prime boosts lexical activation of the target word which then reduces lexical competition and isolates the effects of the second TL prime to sublexical orthographic priming. This difference in masked priming paradigms suggests that the reversed TL priming observed in the deaf readers of our study might arise from lexical-level inhibition effects initiated by the TL prime. There is some evidence for earlier lexical-level competition effects for deaf than hearing readers. Winsler et al. (2023) observed earlier (150 ms post word onset) orthographic neighborhood density effects (greater negativity for words with many neighbors) for deaf compared to hearing readers in a large (unmasked) lexical decision experiment. It is possible that the greater N250 negativity for target words preceded by related TL primes reflects increased activation of sublexical representations from the target word and its neighbors (which is suppressed in the sandwich priming paradigm). We speculate that this neural pattern is unique to deaf readers because of their greater reliance on the visual orthographic code and possibly more robust whole-word orthographic representations.

Emmorey et al. (2021) also reported a reversed N250 priming effect for deaf readers in a masked repetition priming study (greater negativity for repeated than unrelated trials), when the masked primes were short (50 ms). One possible explanation suggested by the authors for this result is that the short duration of the primes gave rise to a type of orthographic competition. Specifically, the use of lower-case prime words (e.g., table) and upper-case target words (e.g., TABLE) could have resulted in two different mismatched *visual* representations, which would be present for both unrelated (space – TABLE) and related (table – TABLE) trials. Critically, on repetition trials, there is still a match between the prime and target at the lexical level, which results in a conflict between the visual form level (different representations) and the lexical level (same representation). This visual form/lexical competition may have resulted in a reversed priming effect for the deaf readers. Again, this neural pattern may be unique to deaf readers because they rely more on the visual code when reading, such that they retain more information about case identity or may represent this information more robustly compared to hearing readers (see also Gutierrez-Sigut et al., 2022). Thus, deaf readers exhibit greater sensitivity to the visual mismatch between stimuli formed from lower-case and the same upper-case letters.

Interestingly, both the present study and the Emmorey et al. (2021) study found a significant correlation between the amplitude of the reversed N250 effect and phonological ability in deaf readers, but in opposite directions across the two studies. Specifically, in the present study, deaf readers with better phonological abilities exhibited a larger reversed TL priming effect, whereas in the Emmorey et al. (2021) study, deaf readers with better phonological abilities (assessed with the same tests) exhibited a smaller reversed repetition priming effect. We suggest that the different direction of the correlations may arise from the different levels of competition effects that we hypothesize. Specifically, deaf readers with stronger phonological skills may have more robust whole-word phonological representations, which would lead to more lexical-level neighbor competition that we hypothesize is associated with the reversed TL priming effect. In contrast, we hypothesize that the reversed repetition priming effect is associated with competition between visual form (different cases) and lexical (same) representations. In this case, deaf readers with better phonological skills may develop stronger abstract letter coding since both upper- and lower-case letters map to the same sound. Thus, letter case identity may be less robustly represented or retained less for these deaf readers such that they exhibit smaller visual form/lexical competition effects. Overall, these patterns of results suggest that phonological knowledge can modulate how strongly deaf readers rely on the orthographic code and/or the mapping between sublexical and lexical level representations.

In the N400 epoch, both deaf and hearing readers exhibited typical PH and TL priming (reduced negativities for targets with PH/TL primes compared to their control primes). Pseudohomophone priming for deaf readers in the N400 window is consistent with the results of Gutierrez-Sigut et al. (2017), but contrasts with those of Costello et al. (2021). As suggested in the introduction, one possible explanation for these different results is that deaf readers may be better able than hearing readers to strategically suppress phonological processing when performing an unmasked lexical decision task, whereas masked pseudohomophone primes are not subject to such strategic effects. Overall, parallel N400 priming effects for deaf and hearing readers indicates that the neural differences we observed between these groups occur during sublexical processing and/or at the interaction between sublexical and lexical processing.

In summary, the current work extends the original groundbreaking masked priming study in hearing university students of Grainger et al. (2006) to a population of older non-student deaf and hearing readers. The results, particularly the reversed N250 TL but not PH priming in deaf readers points to the sensitivity of this paradigm for examining the microstructure of the temporal dynamics of visual word processing and bodes well for future studies using variations of this approach for studying individual differences in visual word processing. In the current context we speculate that phonological knowledge modulates how strongly deaf readers rely on whole-word orthographic representations and/or the mapping from sublexical to lexical representations.

## Data Accessibility Statement

The average ERP data used in the analyses are available at: https://osf.io/p3w7q/.

## Footnotes

Fingerspelling in ASL encodes English words using handshapes that correspond to the letters of the alphabet.
